# Comparative efficacy and safety profile for the treatment of humeral bone cysts in children: curettage and mixed bone grafting either with or without elastic intramedullary nailing

**DOI:** 10.1186/s13018-020-02130-6

**Published:** 2021-04-06

**Authors:** Xuan Wang, Jiuhui Han, Yazhou Li, Yuchang Liu, Junzhong Luo

**Affiliations:** Department of Pediatric Orthopedics, The Third Hospital of Hebei Medical University, No. 139 Ziqiang Road, Qiaoxi District, Shijiazhuang, 050051 Hebei China

**Keywords:** Curettage and mixed bone grafting with elastic intramedullary nailing, Curettage and mixed bone grafting, Children with humeral bone cyst, Efficacy, Safety

## Abstract

**Purpose:**

The primary aim of our study was to evaluate the comparative efficacy and safety profile of curettage and mixed bone grafting without instrument or with elastic intramedullary nailing in the treatment of humeral bone cyst in children.

**Methods:**

Our retrospective study included a total of 48 children harboring humeral bone cyst in our hospital from August 2012 to February 2019. The patients enrolled were divided into elastic nailing group with the application of elastic intramedullary nailing (*n* = 25) and control group without using instrument (*n* = 23) during the management of curettage and mixed bone grafting. The following medical outcomes of the two groups were monitored and recorded: the amount of intraoperative blood loss, operation time and postoperative full weight-bearing time, in addition to postoperative clinical effects after 1 year, the function and pain level of shoulder joint before and 1, 3, 6, 9, 12, and 16 months after operation. Follow-up radiographic outcomes were reviewed to observe bone healing, local recurrence and internal fixation loosening, and other postoperative complications.

**Results:**

The clinical curative effect of the elastic nailing group was higher than that of the control group 16 months after operation (96.00% > 73.91%, *P* < 0.05). The intraoperative blood loss and postoperative full weight-bearing time in the elastic nailing group were less than those in the control group (*P* < 0.05), but the operation time was statistically insignificant between the two groups (*P* > 0.05). Before operation, the shoulder joint function of the two groups was comparable (*P* > 0.05), while the function showed remarkably better outcome in the elastic nailing group than control group 1 to 16 months after operation (*P* < 0.05). Before operation, the pain level of the two groups was comparable (*P* > 0.05), while 1 to 16 months after operation, the pain level of the elastic nailing group was significantly lower than that of the control group (*P* < 0.05). Patients in both groups were followed up for 16 months. Mixed bone grafting fusion was indicated by imaging CT and X-ray during the follow-up period, with an average fusion time of 11.3 ± 1.2 months (range, 8–16 months). Three months after operation, there was 1 case of incomplete pathological fracture in the control group, while no related complications occurred in the elastic nailing group. Moreover, no tumor recurrence was observed in the two groups. The two groups were comparable in terms of the incidence of complications (*P* > 0.05).

**Conclusion:**

Children with humeral cyst treated with curettage and mixed bone grafting with the additional use of elastic intramedullary nailing exerted superior results to those without using instrument as there are beneficial outcomes and safety profile and no complications.

## Introduction

Humeral bone cyst is a benign tumor-like lesion, affecting an increasing number of children and adolescents. This type of lesion originates from intramedullary and concealed, which shows expansive growth. Indeed, it can interfere and destroy the formation of normal bone, reduce bone strength, and induce pathological fractures [1, 2]. The current treatments for humeral cyst rely on hormone or bone marrow injection [3], focus elastic intramedullary nailing implantation [4, 5], curettage and mixed bone graftin g[5], and hollow nail drilling and drainage [6]. However, the major challenge with these approaches is the lack of unified standard, and the effects of different treatment methods vary, which leads to negative effect on the quality of life of patients, as well as the prognosis. The most used therapies include focal curettage and mixed bone grafting with or without additional use of elastic intramedullary nailing. But based on the studies of Yao and Ye [7] and Flont et al. [8], the healing rate showed different effects with and without elastic intramedullary nailing during the treatment of focal curettage and mixed bone grafting. The valid and effective intervention for humeral bone cyst is pivotal for patients as it improves the therapeutic effect, quality of life, as well as the prognosis of patients. Regarding the fact that there are limited domestic studies on the efficacy of elastic intramedullary nailing combined with focal curettage and mixed bone grafting, our study attempts to explore the efficacy and safety of elastic intramedullary nailing during focal curettage and mixed bone grafting for the treatment of humeral cyst in children, in order to provide an effective way of treating the disease.

## Data and methods

### Clinical background

**Fig. 1:**
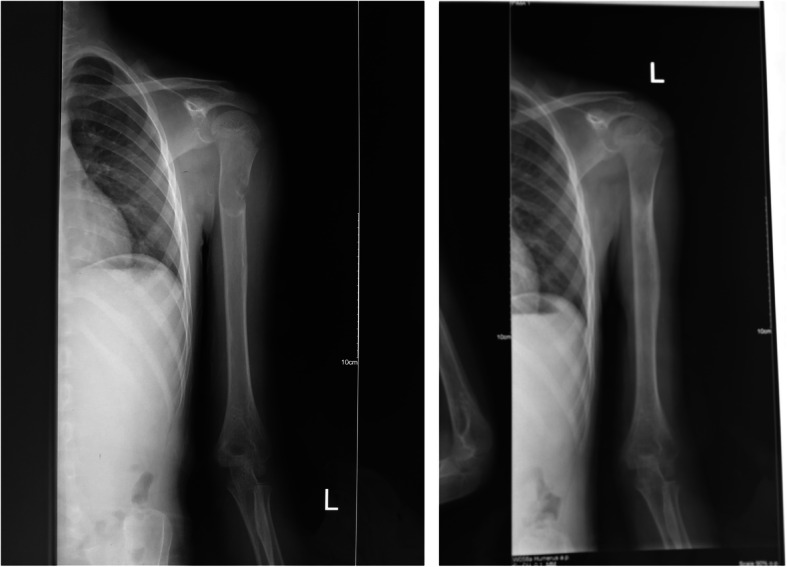
Patients included in this study often presented with local pain or pathological fractures

Our retrospective study included a total of 48 children harboring humeral bone cyst in our hospital from August 2012 to February 2019. The patients enrolled were divided into elastic nailing group with the application of elastic intramedullary nailing (*n* = 25) and control group without using instrument (*n* = 23) during the management of curettage and mixed bone grafting. Children in the elastic nailing group [18 males, 7 females; median age = 10.8 ± 1.0 years (range, 7–16 years)] included 8 cases with lesion on the left side and 17 cases on the right side. Children in the control group [16 males, 7 females; median age = 10.9 ± 1.1 years (range, 7–17 years)] included 6 cases with the lesion on the left side and 17 cases on the right side. Patients included in this study often presented with local pain or pathological fractures (Fig. 1). Fourteen patients had pathological fractures before surgery in the elastic nailing group, while 13 patients in the control group. There was no significant difference in general clinical data between the two groups (*P* > 0.05). The formulation of this research program is in line with the relevant requirements of the Helsinki Declaration of the World Medical Association.

### Selection criteria

Inclusion criteria are as follows: (1) complete clinical data, (2) all patients were firstly treated and with benign pathological results, (3) no disturbance of coagulation system, (4) normal mental state and good compliance of children and their families, (5) ineffective conservative treatment for enrolled patients, (6) patients signed informed consent when the retrospective evaluation was initiated. Exclusion criteria are as follows: (1) patients with severe osteoporosis; (2) patients with serious heart, liver, and kidney diseases; (3) patients without follow-ups; (4) patients with pulmonary hypertension; (5) patients with pulmonary dysplasia and immune system diseases.

### Methods

**Fig. 2:**
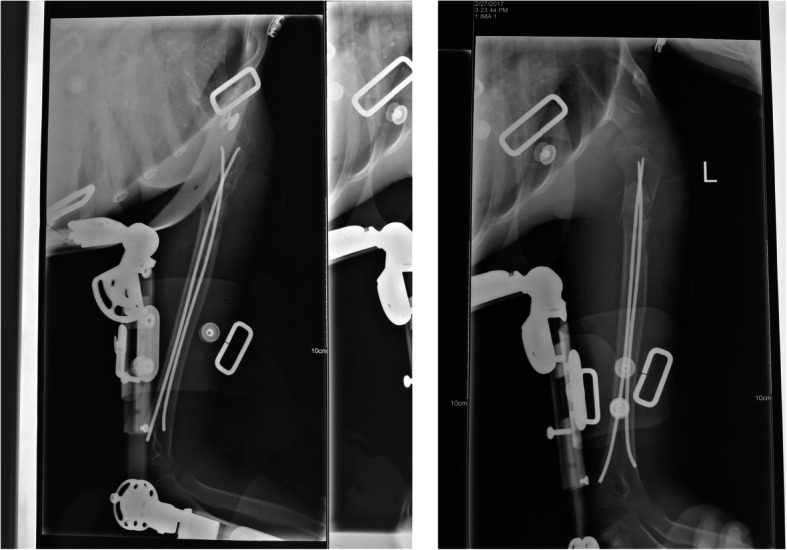
The periosteum and wound (including iliac bone) were sutured layer by layer

Children in elastic nailing group were treated with curettage and mixed bone grafting with additional use of elastic intramedullary nailing. The detailed process was as follows: after general anesthesia with tracheal intubation, affected limb was in the abduction angle, and we performed the routine disinfection and spread the sterile towel sheet. C-arm localization, incision of the skin, subcutaneous and deep fascia at the focus, peeling off the periosteum, preservation of the cortex of the lesion, and drilling and opening of the strip bone window were respectively made and performed. The lesion tissue on the cyst wall was scraped as far as possible without hurting the epiphysis, and the scraped lesion tissue was taken for biopsy. Ninety-five percent anhydrous alcohol was injected into the cyst, for 3 min inactivation. Then, the cyst was rinse repeatedly with povidone iodine and normal saline. A 1–2-cm incision was made in the medial and lateral humerus; the bone malleolus was opened, and a preflex elastic intramedullary nailing (titanium nail) was retrogradely placed in the proximal humerus. The placement of the elastic nail was observed by C-arm X-ray to avoid injury and penetration of the cortex and epiphysis, and the tail nail was treated. One milliliter bone marrow was taken from one side of the iliac spine with a bone puncture needle and injected into the focus of the bone cyst. After the original lesion was curetted, allogeneic bone (Jinwei bone, XKC Medical Technology Development Co., Ltd, Beijing, China) and iliac tissue were mixed and implanted into the cyst cavity, with ½ for each part. If the lesion is large, it is only possible to take the ilium tissue for mixed grafting. The bone cavity was filled tightly along the trabecular weight-bearing line, and the bone cortex window was inactivated with 95% anhydrous alcohol. After repeated rinsing with normal saline, it was replanted to the place where the window was opened. The periosteum and wound (including iliac bone) were sutured layer by layer. Then, the affected limb was fixed with external brace at the end of the operation (Fig. 2).

Children in the control group were treated with single focal curettage and mixed bone grafting, except for the steps of elastic intramedullary nailing implantation, the other steps were the same as those in the elastic nailing group.

### Postoperative intervention

Antibiotics were given for 2 days after operation to avoid infection, and low molecular weight heparin was used for anticoagulation until 1 day before discharge. When the drainage volume was less than 50 ml, the drainage tube should be removed. After extubation, the time of walking on the ground was determined according to the degree of bone defect: patients with severe defect began to walk 1 month after operation, and those with mild defect could leave their beds after removal of the drainage tube. Patients gradually changed from partial weight-bearing to full weight-bearing according to the degree of bone healing. The patients with good healing were discharged from hospital 7 days after injury, and those with poor healing were strengthened to change medicine until the wound healed. Because of the high postoperative pathological risk of the disease, reoperation is not necessary if there are no local pain symptoms after operation; if there are local pain symptoms after operation, reoperation is needed.

### Observation indicators

The amount of intraoperative blood loss, operation time, and postoperative full weight-bearing time of the two groups were calculated. The clinical effects of the two groups were observed 12 months after operation. The shoulder joint function and pain were recorded before and 1, 3, 6, 9, 12, and 16 months after operation. CT and X-ray were followed up to observe bone healing, local recurrence and internal fixation loosening, and other postoperative complications.

#### Clinical efficacy

Based on the modified NEER bone cyst evaluation criteria [9], the clinical efficacy could be divided into 4 aspects: (1) cured: the cyst cavity was filled with new bone, the cyst completely disappeared, the cyst wall thickened, and there was no residual light transmission area; (2) effective: the cyst bone cortex was similar to the normal one, and part of the cyst cavity remained; (3) ineffective: there was no significant change in cyst size and cyst wall thickness before and after treatment, so patients needed to be retreated or restricted activity; (4) relapse: the cyst cavity became smaller, and the cyst wall thickened at the beginning of treatment, then the cyst cavity became larger and the cyst wall became thinner. Clinical efficacy = effective rate + recovery rate.

#### Shoulder joint function

The function of shoulder joint was evaluated by NEER score [10]. There were 13 items in the scale, and the full score was 100. The high score represents good function of hip joint.

#### Pain level

Visual analog score (VAS) [11] was used to score pain level. A long ruler of 10 cm was used; 0-cm segment indicates no pain; 10-cm segment indicates unbearable pain.

### Statistical analyses

The SPSS 21.0 software was used to analyze the data. The measurement data were expressed as $\overline{x}\pm s$, repeated measurement variance analysis was used to analyze the overall comparison of each group data, *t* test was applied to compare the inter-group and intra-group data, and the counting data was represented by rate (%), and chi-square χ^2^ test was used for comparison. A *P* < 0.05 represented statistical significant difference.

## Results

### Comparison of clinical efficacy between the two groups

Twelve months after operation, the clinical curative effect of the elastic nailing group was significantly higher than that of the control group (96.00% > 73.91%, *P* < 0.05), as shown in Table 1.

**Table 1:** Comparison of clinical efficacy between the two groups (*n*, %)

Group	*n*	Cured	Effective	Ineffective	Relapse	Efficacy rate
Elastic nailing group	25	9	14	1	0	24 (96.0)
Control group	23	7	10	6	0	17 (73.9)
*χ*^2^ value						4.691
*P* value						0.03

### Comparison of intraoperative blood loss, operation time, and postoperative full weight-bearing time between the two groups

The intraoperative blood loss, postoperative partial weight-bearing time, and postoperative full weight-bearing time in the elastic nailing group were less than those in the control group (*P* < 0.05), but the operation time was statistically insignificant between the two groups (*P* > 0.05), as shown in Table 2.

**Table 2:** Comparison of intraoperative blood loss, operation time, postoperative partial weight-bearing time, and postoperative full weight-bearing time between the two groups ($\overline{x}\pm s$)

Group	*n*	Intraoperative blood loss (ml)	Operation time (min)	Postoperative full weight-bearing time (week)	Postoperative full weight-bearing time (month)
Elastic nailing group	25	732.8 ± 121.1	186.9 ± 12.3	3.2 ± 0.3	3.9 ± 0.5
Control group	23	769.8 ± 132.8	184.0 ± 16.2	3.9 ± 0.4	4.3 ± 0.4
*t* value		− 5.193	0.704	− 6.417	− 4.788
*P* value		< 0.001	0.485	< 0.001	< 0.001

### Comparison of shoulder joint function between the two groups

Before operation, the shoulder joint function of the two groups was comparable (*P* > 0.05), while the function showed remarkably better outcome in the elastic nailing group than control group 1 to 12 months after operation (*P* < 0.05), as laid out in Table 3.

**Table 3:** Comparison of shoulder joint function between the two groups ($\overline{x}\pm s$)

Group	*n*	Before operation	1 month after operation	3 months after operation	6 months after operation	9 months after operation	12 months after operation	16 months after operation
Elastic nailing group	25	66.9 ± 8.7	78.9 ± 7.9	80.3 ± 8.0	81.3 ± 8.3	85.0 ± 8.0	85.9 ± 8.9	86.0 ± 9.0
Control group	23	67.0 ± 8.1	74.8 ± 8.0	75.7 ± 7.4	75.8 ± 7.6	76.3 ± 7.9	79.6 ± 8.0	80.1 ± 10.2
*t* value		− 0.037	2.159	2.315	2.355	3.769	3.786	4.282
*P* value		0.971	0.037	0.025	0.022	< 0.001	< 0.001	< 0.001

### Comparison of pain level between the two groups

Before operation, the pain level of the two groups was comparable (*P* > 0.05), while 1 to 12 months after operation, the pain level of the elastic nailing group was significantly lower than that of the control group (*P* < 0.05), as shown in Table 4.

**Table 4:** Comparison of pain level between the two groups ($\overline{x}\pm s$)

Group	*n*	Before operation	1 month after operation	3 months after operation	6 months after operation	9 months after operation	12 months after operation	16 months after operation
Elastic nailing group	25	3.9 ± 0.2	2.0 ± 0.2	1.7 ± 0.1	1.0 ± 0.2	0.4 ± 0.1	0.3 ± 0.1	0.3 ± 0.1
Control group	23	3.9 ± 0.2	2.4 ± 0.2	2.0 ± 0.2	1.5 ± 0.1	1.1 ± 0.1	0.9 ± 0.1	0.8 ± 0.1
*t* value		0.517	− 8.246	− 6.958	− 11.741	− 28.527	− 24.451	− 34.959
*P* value		0.608	< 0.001	< 0.001	< 0.001	< 0.001	< 0.001	< 0.001

### Follow-up results

**Fig. 3:**
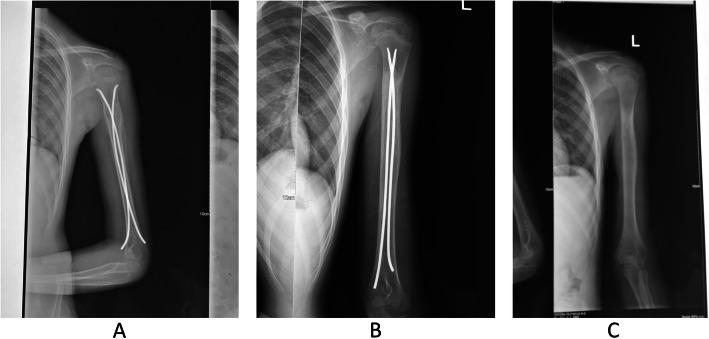
Mixed bone grafting fusion was indicated by imaging CT and X-ray during the follow-up period

Patients in both groups were followed up for 12 months. Mixed bone grafting fusion was indicated by imaging CT and X-ray during the follow-up period (Fig. 3), with an average fusion time of 11.3 ± 1.2 months (range, 8–16 months). Three months after operation, there was 1 case of incomplete pathological fracture in the control group, while no related complications occurred in the elastic nailing group. Moreover, no tumor recurrence and distant metastasis were observed in the two groups. The two groups were comparable in terms of the incidence of complications (*P* > 0.05).

## Discussion

Simple bone cyst is a benign tumor-like lesion that commonly occurred among children, accounting for 3% of all bone tumors [12]. According to the related epidemiological statistical analysis [13], simple bone tumors often occur among children aged 5 to 15, most of them are males. The condition can occur at any part of body bones, especially the proximal femur and humerus. The pathogenesis of humeral bone cyst is unclear; hence, there is a variety of treatment approaches. Conservative treatment is often used in clinical practice, such as bone traction, splint, and plaster support after pathological fracture occurs. Corresponding measures are taken to treat the focus after pathological fracture healing. Minimally invasive treatment includes liquid bone collagen, allogeneic bone powder, and autologous bone marrow [3, 14, 15]. And surgical treatment includes focal curettage and bone grafting and internal fixation implantation and drainage. However, for patients with osteodysplasia, especially those combined with pathological fractures, surgical intervention is the sole option. The conservative treatment is not recommended to treat bone cyst as it can lead to high recurrence rate, even as much as 90% [16].

Focal curettage and mixed bone grafting is currently one of the effective approaches in treating humeral cyst, but the methods fail to show effect in some children [17], possibly due to the fact that most of the lesions near the metaphysis cannot be completely removed to avoid injury to the metaphysis during the operation. It is easy to recur in the later stage; hence, some patients need to seek multiple surgical treatments, and the surgical failure rate is as high as 30% [18, 19]. In addition, considering the expansion and thinning of the local bone cortex of the bone cyst focus, only treatment of focal curettage and mixed bone grafting leads to continuous destruction of the local bone cortex, which is prone to fracture in the process of rehabilitation, improper functional training or minor trauma is easy to cause focus fracture. The study by Komiya et al. [20] confirmed that there are osteoclast inflammatory factors such as interleukin and prostaglandin in the cyst fluid of humeral bone cyst, while elastic intramedullary nailing has the advantages of convenient operation and less trauma. In the treatment of long bone cyst of extremities, the instrument has the effect of continuous drainage of cyst fluid, which can reduce the destruction of cyst fluid to the local bone of the focus, promote the bone marrow cavity to connect with the focus, and then destroy multiple cyst walls. The approach results in reduced local pressure of the focus and improved venous blood flow, which is capable of stabilizing the fracture. Nevertheless, when the elastic intramedullary nailing was implanted, the focus of bone cyst still existed, and the ideal effect of some patients was not achieved. According to the study supported by Shi et al. [13], 26 patients with long bone cysts of extremities were treated with elastic intramedullary nailing, and the cure rate was only 65.4% (17/26). Based on the above features of elastic intramedullary nailing, domestic scholars have applied this instrument with focal curettage and mixed bone grafting in the treatment of long bone cyst, achieving good results.

Elastic intramedullary nailing is now widely used in the treatment of long bones of extremities such as the humerus with lesion occurred in the diaphysis and metaphysis, which exerts favorable patient outcome regardless of the size of cyst and the existence of pathological fracture. According to Abdel-Wanis et al. [21], patients harboring simple bone cyst achieved good clinical effect by using elastic intramedullary nailing; Pogorelić et al. [22] confirmed that elastic intramedullary nailing achieved promising results; Erol et al. [23] and other studies suggested that patients with humeral bone cyst were treated with elastic intramedullary nailing and curettage and mixed bone grafting, and the cure rate was as high as 100%. Based on aggregated results of our study, the clinical effect of the elastic nailing group was better than that of the control group 12 months after operation (*P* < 0.05), which was consistent with that of previous studies, suggesting that focal curettage and mixed bone grafting with the additional use of elastic intramedullary nailing exerted superior outcome to single focal curettage and mixed bone grafting without any instrument in terms of treating humeral bone cyst. However, the treatment failed in 1 case in this study because the bone cyst near the metaphyseal cyst wall was not removed thoroughly in order to protect the epiphysis, resulting in the relapse of the proximal metaphyseal focus after operation. The results of this study demonstrated that the intraoperative blood loss, postoperative partial weight-bearing time, and postoperative full weight-bearing time in the elastic nailing group were significantly less than those in the control group (*P* < 0.05). However, the operation times between the two groups were comparable (*P* > 0.05), indicating that focal curettage and mixed bone grafting with elastic intramedullary nailing led to effective reduction in the intraoperative blood loss and full weight-bearing time after operation. Moreover, the approach was not associated with increased operation time, which is beneficial to the recovery of the children with humeral cyst.

Based on our results, the function of shoulder joint in the elastic nailing group was better than that in the control group 1 to 12 months after operation (*P* < 0.05), and the pain level in the elastic nailing group was lower than that in the control group. It was suggested that the treatment of humeral cyst with elastic intramedullary nailing and curettage combined with mixed bone grafting is beneficial to the recovery of shoulder joint function and reduces the degree of pain. The results may attribute to the fact that the combination of curettage and mixed bone grafting with elastic intramedullary nailing fully decompress and drains the focus, which is able to fix pathological fracture and prevent the occurrence of pathological fracture [24]. Hence, it effectively restores the function of the shoulder joint and relieves the pain, and is conducive to the rehabilitation of patients. Additionally, all children were followed up for 16 months. And the results showed that mixed bone grafting fusion was indicated by imaging CT and X-ray during the follow-up period, and the incidence of complications was comparable between the two groups (*P* > 0.05). It was suggested that the treatment of humeral cyst with elastic intramedullary nailing and focal curettage and bone grafting is beneficial to bone fusion without increasing the incidence of complications with safe profile. The research supported by Donaldson et al. [25] suggested that some patients with bone cyst could be cured, but the local focus still existed for a long time. The humerus has a high risk of fracture under the action of external force as children and adolescents tend to move a lot and are prone to accidents such as falls and trauma; hence, it is always important to achieve the therapeutic effect in the treatment of humeral bone cyst in children and cure them if possible. In this study, the children in the elastic nailing group treated with elastic intramedullary nailing and focal curettage and mixed bone grafting achieved favorable outcomes, the cure rate was high, and there were no complications. However, regarding the retrospective feature of this study, there are several limitations that should be acknowledged. Our study failed to compare the pathological fracture, age, cyst septum, focus size, cyst index, and cyst area. The sample size included in this study is small; hence, more multicenter randomized controlled studies with larger sample size should be warranted to confirm the best treatment and etiology of humeral cyst.

Taken together, elastic intramedullary nailing combined with curettage and mixed bone grafting is effective in the treatment of patients with humeral bone cysts, which elicits beneficial outcomes in terms of reducing the amount of intraoperative blood loss, the time of partial and full weight-bearing after operation, and contributes to the recovery of shoulder joint function, with less pain and low occurrence of complications. Children harboring humeral bone cyst with large focus and pathological fracture or fracture tendency should seek intervention of focal curettage and bone grafting with the use of elastic intramedullary nail.

## Data Availability

The datasets generated and analyzed during the current study are available from the corresponding author on reasonable request.

## Abbreviation

VAS: Visual analog score
